# Pancreatic β cell-secreted factor FGF23 attenuates Alzheimer's disease-related amyloid β-induced neuronal death

**DOI:** 10.1093/pnasnexus/pgae542

**Published:** 2025-01-28

**Authors:** Kyosuke Yazawa, Mieko Nakashima, Tadashi Nakagawa, Yuhki Yanase, Yukari Yoda, Koichiro Ozawa, Toru Hosoi

**Affiliations:** Department of Pharmacotherapy, Graduate School of Biomedical and Health Sciences, Hiroshima University, 1-2-3 Kasumi, Minami-ku, Hiroshima 734-8551, Japan; Department of Pharmacotherapy, Graduate School of Biomedical and Health Sciences, Hiroshima University, 1-2-3 Kasumi, Minami-ku, Hiroshima 734-8551, Japan; Department of Clinical Pharmacology, Faculty of Pharmaceutical Sciences, Sanyo-Onoda City University, 1-1-1 Daigaku-dori, Sanyo Onoda City, Yamaguchi 756-0884, Japan; Department of Pharmacotherapy, Graduate School of Biomedical and Health Sciences, Hiroshima University, 1-2-3 Kasumi, Minami-ku, Hiroshima 734-8551, Japan; Department of Pharmacotherapy, Graduate School of Biomedical and Health Sciences, Hiroshima University, 1-2-3 Kasumi, Minami-ku, Hiroshima 734-8551, Japan; Department of Pharmacotherapy, Graduate School of Biomedical and Health Sciences, Hiroshima University, 1-2-3 Kasumi, Minami-ku, Hiroshima 734-8551, Japan; Department of Clinical Pharmacology, Faculty of Pharmaceutical Sciences, Sanyo-Onoda City University, 1-1-1 Daigaku-dori, Sanyo Onoda City, Yamaguchi 756-0884, Japan

**Keywords:** Alzheimer's disease, amyloid β, pancreatic β cell, ribosomal protein, diabetes

## Abstract

Alzheimer's disease (AD) is a neurodegenerative disorder characterized by cognitive decline and memory impairment. The pathophysiology of AD may involve aggregated amyloid β (Aβ) accumulation, which may underlie the disease mechanism. Patients with diabetes exhibit an elevated risk of developing AD, indicating potential therapeutic implications upon elucidating the underlying mechanisms. We hypothesized that pancreatic β cell-secreted factors could protect neurons from Aβ-induced toxicity. Therefore, we established an experimental model to elucidate the communication between pancreatic β cells and neuronal cells. Notably, our findings demonstrate that pancreatic β cell culture supernatant effectively inhibits Aβ-induced neuronal cell death. Transcriptomic analysis showed significant up-regulation of multiple ribosomal protein genes in neuronal cells treated with pancreatic β cell culture supernatant. Fibroblast growth factor 23, a secreted factor from pancreatic β cells, significantly suppressed Aβ-induced neuronal cell death. Our findings suggest that pancreatic β cells may secrete previously unrecognized neuroprotective factors, thereby attenuating neuronal cell death in AD.

**Significance Statement**

Aggregated amyloid β (Aβ) accumulation in the brain was suggested to cause Alzheimer's disease (AD), a neurodegenerative disorder characterized by cognitive decline and memory impairment. Several brain-derived neurotropic factors for AD treatment have been reported to date. On the contrary, although pancreatic β cell death in patients with diabetes causes AD, the link between peripheral-derived factors and AD is not well understood. Therefore, we hypothesized the possibility that periphery-derived factors may affect neuronal cells and may ameliorate Aβ-induced cell death. In the present study, we discovered that pancreatic β cells secrete a neuroprotective factor, which may lead to translation in neurons. The results suggest that the interaction between pancreatic β cells and neurons may have the potential to improve the progression of AD.

## Introduction

Alzheimer's disease (AD), a neurodegenerative condition characterized by the presence of senile plaques and neurofibrillary tangles, manifests as progressive neuronal loss culminating in profound cognitive decline. Global dementia prevalence exceeds 55 million individuals, with nearly 10 million new cases arising annually (World Health Organization, 2023 March 15). While AD represents the predominant cause of dementia cases, accounting for 60–70%, the precise etiological mechanisms remain elusive. The “amyloid cascade hypothesis” posits that the aggregation of amyloid β (Aβ) instigates aberrant tau protein phosphorylation, leading to neuronal degeneration and disruption of brain homeostasis (1).

Diabetes mellitus is a metabolic disorder characterized by chronic hyperglycemia resulting from insufficient insulin secretion by pancreatic β cells. It is classified into two main types: type 1 and type 2 (2). Type 1 diabetes is considered an autoimmune condition involving genetic, immunological, and environmental factors. Autoimmune attacks on pancreatic β cells lead to their destruction, characterized by inflammatory responses and infiltration by mononuclear cells (3, 4). In contrast, type 2 diabetes has a multifaceted pathogenesis, encompassing various degrees of β cell dysfunction (5). Major risk factors for type 2 diabetes include obesity-related insulin resistance and impaired insulin secretion due to β cell dysfunction (6). Progressive deterioration in β cell function results in glucose intolerance, contributing to the progression of type 2 diabetes. In summary, both type 1 and type 2 diabetes are characterized by diminished pancreatic β cell function.

Intriguingly, an epidemiological analysis has unveiled a notable correlation between diabetes and AD, indicating that patients with diabetes exhibit a relative risk of 1.2–2.3 for developing AD (7). Moreover, experimental findings in murine models indicate a cognitive decline in animals with impaired pancreatic function induced by streptozocin, which selectively targets pancreatic β cells (8). This underscores a potential causal relationship between pancreatic β cell function and the onset of AD. These findings raise the hypothesis that humoral factors released by the pancreas may exert inhibitory effects on AD. Insulin's neuroprotective properties (9) suggest that peripheral pancreatic β cell-derived insulin could modulate neuronal activity to mitigate the onset of AD. However, conflicting reports suggest that insulin alone may not be efficacious in halting the onset of AD (10), implying that β cell-derived insulin alone may not fully elucidate the etiology of AD. Hence, factors derived from peripheral β cells, distinct from insulin, may also play a role in suppressing the onset of AD. Nonetheless, the precise underlying mechanisms remain elusive and warrant further investigation.

Building upon these insights, we hypothesized the presence of a still-unknown neuroprotective agent, distinct from insulin and potentially secreted by pancreatic β cells, that could mitigate the onset of AD. Accordingly, we devised an in vitro model aimed at directly discerning the neuroprotective factors produced by pancreatic β cells. Furthermore, leveraging this experimental framework, we discerned potential candidates for neuroprotective factors originating from β cells and scrutinized their underlying mechanisms of neuroprotection.

## Results

### The supernatant derived from the pancreatic β cell line mitigates neuronal cell death induced by Aβ

Given the heightened risk of AD associated with diabetes, which impairs pancreatic β cells, we sought to explore the potential of these cells to produce neuroprotective factors. To this end, we established an in vitro system capable of assessing neuroprotective factors secreted by pancreatic β cells. In our investigation, we utilized the Min6 cell line as a model for pancreatic β cells (11) and the PC12 cell line as a model for neuronal cells (12).

**Fig. 1.**
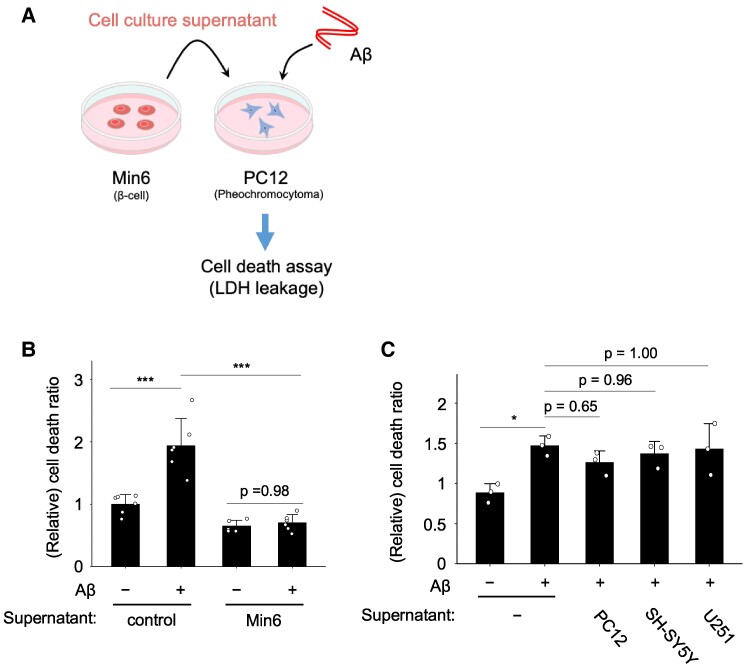
Protection against Aβ-induced neuronal cell death by culture supernatant of pancreatic β cells. A) Schematic representation of the present experimental condition. PC12 neuronal cells were treated with Min6 pancreatic cell culture supernatant, and Aβ-induced neuronal cell death was analyzed. B) PC12 cells were treated with Aβ_1–42_ (5 µM) and Min6 cell culture supernatant. Min6 cell culture supernatant was found to attenuate Aβ-induced neuronal cell death. The percentage of cell death was measured by the LDH assay after 48 h of incubation. C) PC12 cells were cultured with Min6, PC12, SH-SY5Y, or U251 cell culture supernatant, and Aβ_1–42_ (5 µM)-induced cell death was analyzed. Only Min6 pancreatic β cell line culture supernatant showed a neuroprotective effect against Aβ_1–42_-induced cell death. The percentage of cell death was measured by the LDH assay after 48 h of incubation. Mean ± SE, B) *n* = 6 and C) *n* = 3. *P < 0.05; ***P < 0.001. Tukey–Kramer test.

To evaluate whether the culture supernatant of Min6 cells exhibits a neuroprotective effect against Aβ-induced cell death, PC12 neuronal cells were exposed to Min6 cell culture supernatant and subjected to Aβ treatment, followed by cell death analysis using the lactate dehydrogenase (LDH) leakage assay (Fig. 1A). Several forms of Aβ exist, with Aβ_1–42_ demonstrated to be the most relevant to disease pathogenesis (13, 14); therefore, it was used in this investigation. In the absence of Min6 cell culture supernatant, Aβ_1–42_ treatment increased PC12 cell death, indicative of the sensitivity of PC12 cells to Aβ_1–42_-induced cell death (Fig. 1B), as previously reported (15, 16). The presence of Min6 cell culture supernatant significantly attenuated this cell death (Fig. 1B), suggesting a neuroprotective effect against Aβ_1–42_ toxicity. To assess whether this neuroprotective effect was specific to the Min6 pancreatic β cell line, we examined the effects of culture supernatants from other cell lines, including PC12 neuronal cells, SH-SY5Y neuroblastoma cells, and U251 glioma cells, on Aβ_1–42_-induced cell death in PC12 cells. We did not observe a neuroprotective effect with these culture supernatants, implying that the neuroprotective effect was unique to the Min6 pancreatic β cell culture supernatant (Fig. 1C). These findings suggest that a specific factor present in the culture supernatant of the Min6 pancreatic β cell line may exert a neuroprotective effect against Aβ_1–42_-induced cell death.

### Insulin exhibited no discernible impact on Aβ-induced neuronal cell death

**Fig. 2.**
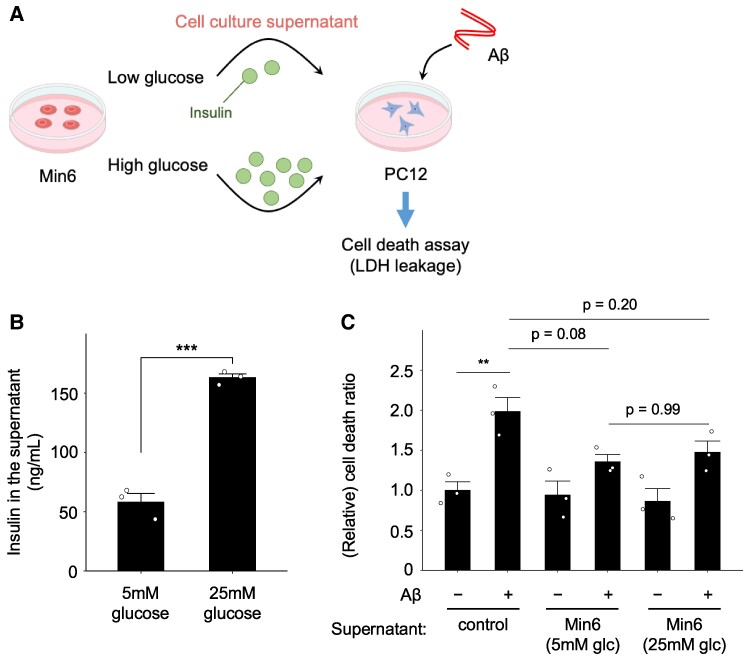
The neuroprotective effect against Aβ toxicity was irrelevant to insulin produced by pancreatic β cells. A) Schematic representation of the current experimental conditions. PC12 neurons were treated with Min6 pancreatic cell culture supernatant, and Aβ-induced neuronal cell death was analyzed. High glucose increased insulin production in Min6 cell culture supernatant. Then, the supernatant was subjected to PC12 neuronal cells and analyzed for Aβ-induced cell death. B) Insulin secreted from Min6 cells was measured by ELISA. Min6 cells were cultured in KRH buffer (5 mM glucose and 25 mM glucose) for 4 h. Mean ± SE, *n* = 3. ***P < 0.001. Student's t test. C) Min6 pancreatic β cell lines were cultured in KRH buffer (5 mM glucose) for 30 min. Then, the medium was replaced with KRH buffer containing 25 mM glucose and cultured for 4 h. The Min6 cell culture supernatant was filtrated and replaced with neurobasal medium (B-27 antioxidant-free). PC12 cells were treated with the medium and stimulated with Aβ for 48 h. The LDH activity of cells and culture medium was then measured as an indicator of cytotoxicity. Data were expressed as (medium/[medium + cell]) and analyzed as fold intensity compared with control samples. Mean ± SE, *n* = 3. **P < 0.01; ***P < 0.001. Tukey–Kramer test.

The Min6 pancreatic β cell line triggers insulin secretion in response to glucose stimulation (17). Given that insulin has been documented to exert a neuroprotective effect against Aβ (18), the insulin released from Min6 cell cultures may have mitigated Aβ-induced cell demise. To explore this prospect, we examined Aβ-induced neuronal cell death using Min6 cell culture medium exposed to glucose (Fig. 2A). Initially, we assessed whether glucose influenced insulin production in Min6 cells. To this end, these cells were incubated with two varying glucose concentrations: 5 or 25 mM glucose, and insulin levels were quantified by Enzyme-Linked Immunosorbent Assay (ELISA) (Fig. 2B). As previously reported (17), a significant increase in insulin secretion was observed with 25 mM glucose compared with 5 mM glucose (Fig. 2B). We then investigated whether the glucose-induced insulin in Min6 cell culture supernatant impacted Aβ_1–42_-induced cell death. PC12 cells were treated with the supernatants of Min6 cells preincubated with 5 or 25 mM glucose, and Aβ_1–42_-induced cell death was assessed (Fig. 2A). No differences could be discerned between 5 and 25 mM glucose on the protective effect of Min6 cells against the neuronal cell death (Fig. 2C). These findings suggest that Min6 cell culture supernatant harbors neuroprotective factors distinct from insulin.

### The neuroprotective factors present in the pancreatic β cell culture medium are thermolabile

**Fig. 3.**
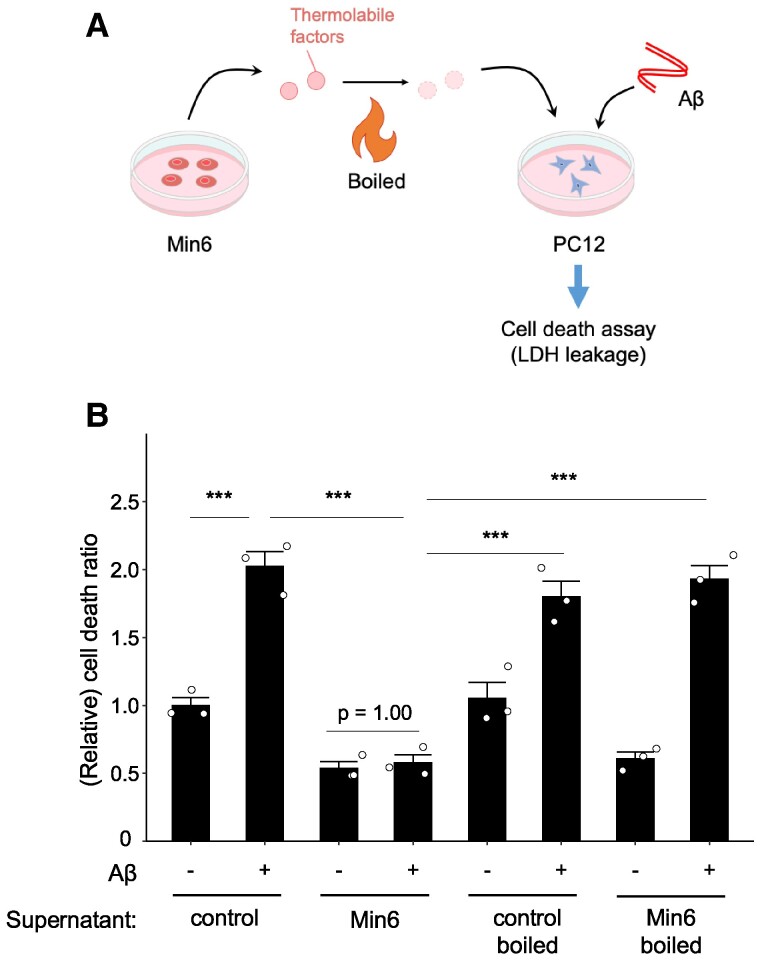
The neuroprotective effect of Min6 cell culture supernatant was attenuated by boiling. A) Schematic representation of the current experimental conditions. PC12 neurons were treated with Min6 pancreatic cell culture supernatant, which was boiled for 10 min, and Aβ-induced neuronal cell death was analyzed. Thermolabile factors were inactivated by boiling the supernatant. B) Min6 pancreatic β cell lines were cultured with neurobasal medium (B-27 antioxidant-free) for 16 h. Then, the culture supernatant was heated at 100 °C for 10 min. The supernatant was transferred to PC12 cells and cultured for 48 h with or without Aβ. The LDH activity of cells and culture medium was measured as an indicator of cytotoxicity. Data were expressed as (medium/[medium + cell]) and analyzed as fold intensity compared with control samples. Mean ± SE, *n* = 3. ***P < 0.001. Tukey–Kramer test.

To address the factors responsible for neuroprotection, we conducted physicochemical analyses to evaluate the nature of these potential neuroprotective substances. Specifically, we investigated the stability of these factors by boiling the Min6 cell culture supernatant for 10 min and then analyzing its protective effect against Aβ_1–42_-induced neuronal cell death (Fig. 3A). Boiling the Min6 cell culture supernatant eliminated its protective effect against Aβ_1–42_-induced neuronal cell death (Fig. 3B). These results support the notion that the neuroprotective factors present in the Min6 pancreatic β cell culture supernatant are thermolabile.

### The supernatant derived from pancreatic β cell culture exhibited a neuroprotective effect against the toxicity induced by aggregated Aβ

**Fig. 4.**
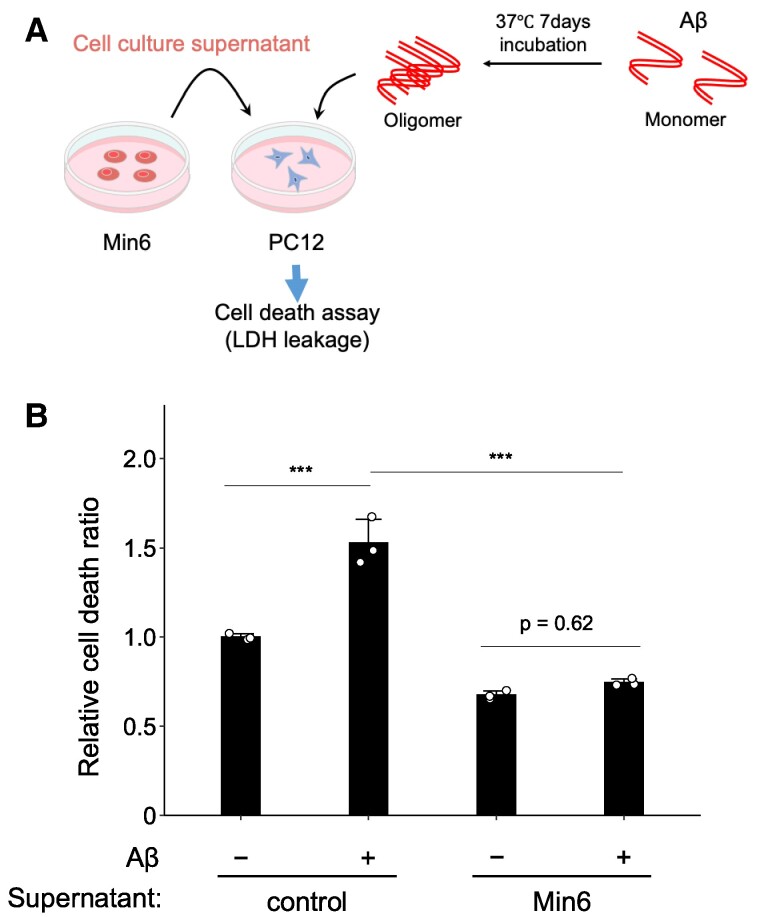
Protection against aggregated Aβ-induced neuronal cell death by culture supernatant of pancreatic β cell. A) Schematic representation of the current experimental conditions. PC12 neurons were treated with Min6 pancreatic cell culture supernatant, and Aβ-induced neuronal cell death was analyzed. Aβ_25–35_ was incubated at 37 °C for 7 days to form aggregate before the treatment. B) Aβ_25–35_ was preincubated at 37 °C for 1 week. PC12 cell culture supernatant was replaced with Min6 cell culture supernatant and then stimulated with Aβ_25–35_ (20 µM). Cell death was measured by the LDH assay after 48 h of incubation. Min6 cell culture supernatant was found to attenuate preaggregated Aβ-induced neuronal cell death. Mean ± SE, *n* = 3. ***P < 0.001. Tukey–Kramer test.

To ascertain whether the pancreatic Min6 cell culture supernatant conferred neuroprotection against the aggregated form of Aβ toxicity, we exposed PC12 neuronal cells to aggregated Aβ_25–35_ in the presence or absence of Min6 cell culture supernatant and examined cell death (Fig. 4A). Of note, Aβ_25–35_ is the shortest toxic fragment of the full-length peptide Aβ_1–42_ and represents the biologically active region of Aβ (19). It has also been reported that Aβ_25–35_ can aggregate from a soluble monomer into a fibrillar β-sheet structure similar to that of Aβ_1–42_ (20). Therefore, Aβ_1–42_ and Aβ_25–35_ demonstrated to have similar biophysical properties, and Aβ_25–35_ has been utilized as a surrogate of Aβ (21, 22). The release of LDH from aggregated Aβ_25–35_-treated PC12 cells without Min6 supernatant was elevated compared with the non-Aβ-treated group (Fig. 4B), indicative of the sensitivity of PC12 cells to Aβ_25–35_-induced cell death, as previously reported (15, 16, 23). The cell death was significantly reduced in the presence of Min6 pancreatic β cell culture supernatant (Fig. 4B), indicating that Min6 supernatant protects PC12 cell death from aggregated Aβ.

### Ribosomal protein genes are up-regulated in response to pancreatic β cell culture supernatant in neuronal cells

**Fig. 5.**
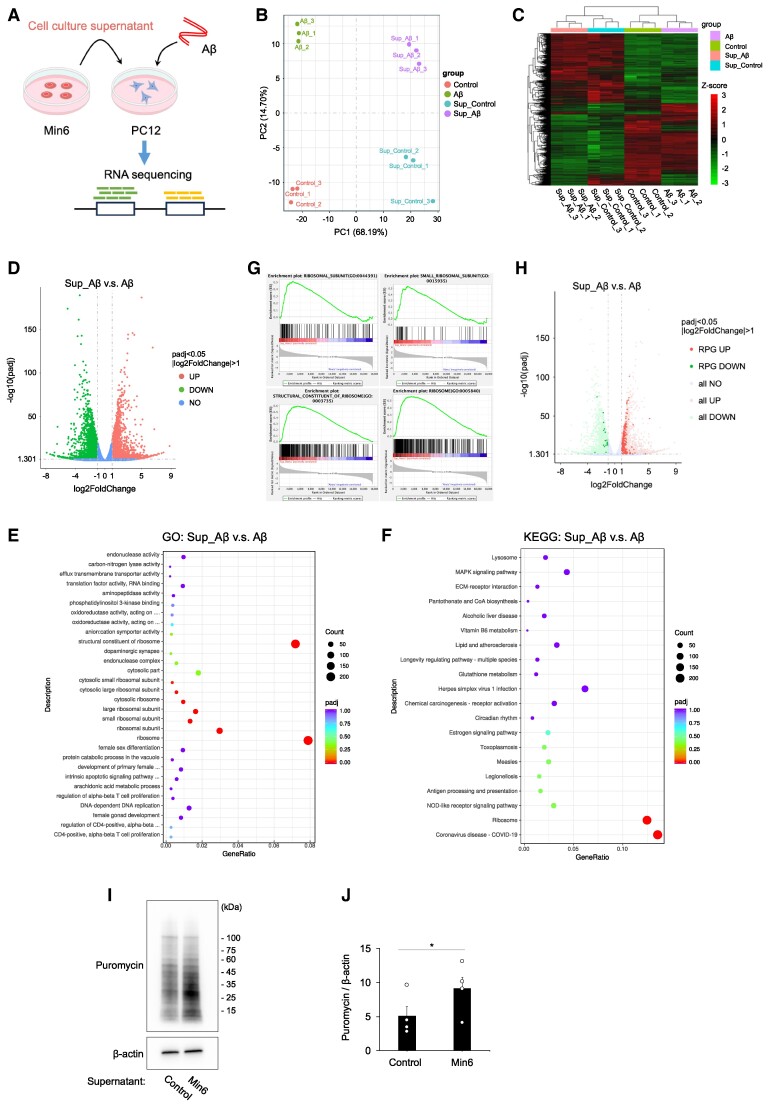
Culture supernatant of pancreatic β cell up-regulated ribosomal protein genes in PC12 neuronal cells. A) Schematic representation of the present experimental condition. PC12 neuronal cells were treated with Min6 pancreatic cell culture supernatant and then stimulated with Aβ. RNA-seq analysis was performed on PC12 neuronal cells. Transcriptome sequencing was performed for four groups, i.e. control samples (Control), Aβ-treated samples (Aβ), Min6 cell culture supernatant-treated samples (Sup_Control), and Min6 cell culture supernatant-treated samples stimulated with Aβ (Sup_Abeta). All groups had three biological replicates. B) PCA of the RNA-seq expression data from PC12 cells at each group. C) Heatmap analysis of each sample. D) Volcano plot of DEGs in PC12 Sup_Abeta vs Abeta. E) GO analysis of DEGs in PC12 Sup_Abeta vs Abeta. F) KEGG pathway analysis of DEGs in PC12 Sup_Abeta vs Abeta. G) Enrichment plot of RIBOSOMAL_SUBUNIT (GO: 0044391), SMALL_RIBOSOMAL_SUBUNIT (GO: 0015935), STRUCTURAL_CONSTITUENT_OF_RIBOSOME (GO: 0003735), and RIBOSOME (GO: 0005840). H) Volcano plot showing DEGs of the ribosomal protein gene. I) Representative images of western blots measuring translational activity. J) Quantitative western blot analysis of translational activity. Mean ± SE, *n* = 4. *P < 0.05. Paired t test.

To further elucidate the underlying mechanisms of neuroprotection, transcriptomic analysis (RNA-seq) was conducted on neuronal cells treated with or without pancreatic β cell culture supernatant in the presence or absence of aggregated Aβ_25–35_ (Fig. 5A). Utilizing the Fragments Per Kilobase of exon per Million mapped reads (FPKM) values of all expressed genes, principal component analysis (PCA) was performed to ascertain the overall expression pattern of the samples. The top two components (PC1 and PC2) revealed distinct separation among the four groups: control samples (Control), Aβ-treated samples (Aβ), Min6 cell culture supernatant-treated samples (Sup_Control), and Min6 cell culture supernatant-treated samples stimulated with Aβ (Sup_Abeta) (Fig. 5B). Furthermore, heatmap data corroborated the distinct gene expression trends observed in the RNA-seq analysis of all samples (Fig. 5C). Hence, the findings indicate that treatment with Aβ and/or Min6 cell culture supernatants could induce alterations in the transcriptome profile of PC12 cells.

To clarify the intracellular changes in PC12 cells treated with min6 cell supernatant, an analysis of differentially expressed genes (DEGs) was conducted, with adjustments made for *P*-values <0.05 and absolute values of log2 fold changes >1. The volcano plot depicting DEGs revealed that Min6 cell culture supernatant-treated samples stimulated with Aβ (Sup_Aβ) vs Aβ-treated samples (Aβ) exhibited 2,187 up-regulated and 2,077 down-regulated DEGs (Fig. 5D). A similar trend was observed in Sup_Control vs Control (Supplementary Figure S1). To elucidate the functional roles of DEGs, Gene Ontology (GO) and Kyoto Encyclopedia of Genes and Genomes (KEGG) pathway analyses were performed. GO analysis indicated the enrichment of ribosome-related terms, including ribosomal subunits, cytosolic ribosomes, and structural constituents of the ribosome, among others, in PC12 neuronal cells (Fig. 5E). Moreover, KEGG analysis revealed enrichment of pathways such as coronavirus disease—COVID-19 and ribosomes in PC12 neuronal cells (Fig. 5F). Gene set enrichment analysis (GSEA) also showed the enrichment of RIBOSOMAL_SUBUNIT (GO: 0044391), SMALL_RIBOSOMAL_SUBUNIT (GO: 0015935), STRUCTURAL_CONSTITUENT_OF_RIBOSOME (GO: 0003735), and RIBOSOME (GO: 0005840) (Fig. 5G). Notably, among the DEGs associated with ribosomal-related GO terms, 189 genes were up-regulated and 18 were down-regulated (Fig. 5H). In summary, treatment with Min6 cell culture supernatant significantly up-regulated the expression of ribosomal protein genes in PC12 cells. Furthermore, as measured by puromycin antibody labeling, ribosomal translational activity was increased in PC12 cells treated with Min6 cell culture supernatant (Fig. 5IJ). These findings characterize the effects of factors contained in Min6 cell culture supernatants on neuronal cells and suggest that the up-regulation of ribosomal genes and the associated increase in translational activity may serve a pivotal role in promoting the survival of neuronal cells against Aβ toxicity.

### Identification of FGF23 as a potential candidate involved in neuroprotection

**Fig. 6.**
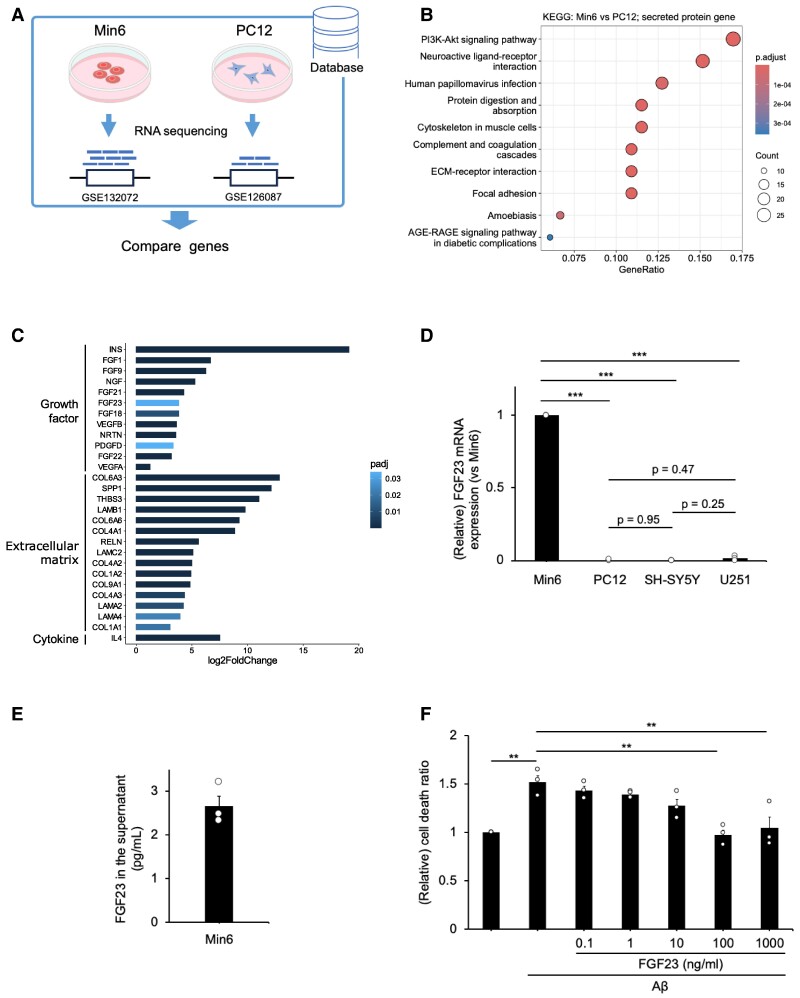
FGF23 was identified as a pancreatic β cell-derived secreted neuroprotective factor. A) Schematic representation of the present experimental condition. Min6 and PC12 RNA-seq data were obtained from a public database and then compared with genes involved in secreted proteins. B) KEGG pathway analysis of secreted protein DEGs in Min6 vs PC12. C) DEGs of PI3K–AKT signaling pathway activator, secreted from Min6 cells. D) Expression levels of *FGF23* mRNA in Min6, PC12, SH-SY5Y, and U251 cells. Mean ± SE, *n* = 3. ***P < 0.001. Tukey–Kramer test. E) FGF23 secreted from Min6 cells was measured by ELISA. F) PC12 cells were treated with Aβ_25–35_ (20 µM) and FGF23 (0.1, 1, 10, 100, and 1,000 ng/ml). The percentage of cell death was measured by LDH assay 48 h after incubation. Mean ± SE, *n* = 3. **P < 0.01. Tukey–Kramer test.

Given the thermolabile nature of the observed neuroprotective factors (Fig. 3B), we speculated that the released factors from pancreatic β cells may be proteins not expressed in PC12 cells. To investigate neuroprotective factors of pancreatic Min6 cell culture supernatant, we analyzed the transcriptome data of Min6 and PC12 cell lines (Fig. 6A). mRNA expression data for Min6 cells (GSE132072) and PC12 cells (GSE126087) were obtained from the genomic database Gene Expression Omnibus (GEO). Genes were filtered, which only included secreted proteins, using the Human Protein Atlas. Among these filtered genes, highly expressed genes at Min6 cells compared with PC12 cells were further analyzed by KEGG pathway analysis using the clusterProfiler R package. The KEGG analysis revealed enrichment of phosphoinositide 3-Kinase (PIK3)–akt serine/threonine kinase (AKT) pathway and its activating proteins secreted from the cells (Fig. 6BC). PI3K–AKT–mammalian target of rapamycin (mTOR) signaling pathway is well known to function as ribosomal protein biogenesis (24). Therefore, activator proteins of the PI3K–AKT pathway, secreted from Min6 cells (Fig. 6C), may attenuate Aβ-induced neuronal death. In the present study, our data analysis indicated that Min6 cell culture supernatant contains multiple growth factors (Fig. 6C). Among them, we focused on fibroblast growth factors (FGFs), which activate PI3K–AKT signaling pathway (25).

Although the association between FGFs and AD has been reported (26), the effect of fibroblast growth factor 23 (FGF23) on AD has not yet been studied. As FGF23 is an endocrine protein (27), we hypothesized that FGF23 is one of the factors that attenuate Aβ-induced PC12 cell death. To test the expression of FGF23 mRNA, we conducted real-time PCR. Compared with the expression of FGF23 in Min6 cells, it was barely detectable in PC12, SH-SY5Y, and U251 cells (Fig. 6D). In contrast to FGF23, the expression levels of FGF1, FGF18, and FGF21 were lower in Min6 cells compared with other cell lines such as PC12, SH-SY5Y, and U251. Therefore, we excluded them from candidate factors (Supplementary Figure S2). The presence of FGF23 protein in the Min6 cell culture supernatants was confirmed using ELISA (Fig. 6E). To ascertain whether FGF23 conferred neuroprotection against the Aβ toxicity, we treated PC12 neuronal cells with aggregated Aβ_25–35_ in the presence or absence of FGF23 (0.1, 1, 10, 100, and 1,000 ng/ml) and examined cell death (Fig. 6F). The cell death was significantly reduced in the presence of FGF23 (Fig. 6F). Similar to the Min6 cell culture supernatant, treatment with FGF23 (100 ng/ml) also increased the translational activity of PC12 cells (Supplementary Figure S3). These results indicate that FGF23 protects PC12 cell death from Aβ toxicity.

## Discussion

Research into the pathogenesis and therapeutics of AD represents an ongoing global endeavor. One promising strategy involves mitigating the toxicity of the harmful peptides Aβ and tau protein. Numerous neuroprotective factors capable of inhibiting Aβ toxicity have been identified (28) such as nerve growth factor, brain-derived neurotrophic factor, and glial cell line-derived neurotrophic factor (29), all of which are brain-derived. However, focusing solely on these brain-derived factors may pose challenges in fully elucidating the mechanisms underlying AD, as peripheral factors also play a role in its pathogenesis (30). Furthermore, diabetes, characterized by a deficit in pancreatic β cells, is associated with an increased risk of AD. Hence, peripheral organ-derived factors may also contribute to the pathogenesis of AD. In light of studies exploring the impact of peripheral factors on brain function, efforts have especially been made to investigate the effects of pancreatic secretory factors on brain function (31, 32).

In the present study, we established an in vitro model system termed the “neuron-pancreatic β cells model system” to explore neuroprotective factors by examining the interplay between pancreatic β cells and neurons. Our findings demonstrate that the culture supernatant of Min6 cells, a model cell line for pancreatic β cells, shields PC12 neuronal cells from Aβ neurotoxicity (Fig. 1). Additionally, the Min6 cell culture supernatant inhibits cell death induced by aggregated Aβ (Fig. 4). Furthermore, we observed that this protective effect against Aβ toxicity is specific to pancreatic β cells (Fig. 1). Therefore, defective neuroprotective factors secreted by the pancreas due to diabetes may impact brain function, rendering individuals more susceptible to Aβ-induced neurotoxicity and AD.

Insulin has been documented to mitigate neuronal cell death induced by Aβ (33). Studies have shown that insulin treatment in hippocampal cells suppresses Aβ-induced neuronal cell death (18). Moreover, treatment with Aβ followed by insulin administration effectively suppressed neuronal cell death, involving caspase-9 inhibition and Hsp70 induction (34). Overall, these findings suggest that the suppressive effect of Min6 cell culture supernatant on Aβ-induced cell death may be mediated through insulin production. However, conflicting observations have been reported, suggesting that insulin and insulin signaling may exacerbate AD (35, 36). In our study, we demonstrated that the inhibitory effect of Min6 cell culture supernatant on PC12 cell death remained unchanged under conditions that induce insulin production (Fig. 2). Therefore, these results suggest that pancreatic β cells may secrete neuroprotective factors other than insulin.

The mechanism underlying the observed neuroprotection by treating pancreatic β cell supernatant remains to be elucidated. We postulated that the Min6 cell culture supernatant might mitigate Aβ-induced PC12 cell death by augmenting survival factors in PC12 cells, thereby shielding them from Aβ toxicity. To explore this hypothesis, we conducted an analysis using RNA-seq to elucidate changes in the transcriptome of PC12 cells treated with Min6 cell culture supernatant. Surprisingly, GO enrichment and KEGG pathway analysis revealed a significant enrichment of genes encoding ribosomal proteins in PC12 cells treated with the Min6 cell culture supernatant (Fig. 5). These findings suggest that the secretory protein population contained in Min6 cell culture supernatant may have characteristics that lead to the up-regulation of ribosomal components in neuronal cells.

Ribosomes are vital intracellular organelles essential for protein translation. Remarkably, studies have revealed associations between ribosome production disorders and defects in various neurodegenerative diseases, including AD, Huntington's disease, Parkinson's disease, amyotrophic lateral sclerosis, and frontotemporal lobe dementia (37). In the early stages of AD, inhibition of protein synthesis mediated by abnormalities in ribosomal RNAs and polyribosome complexes has been documented (38, 39). Our current investigation discovered that pancreatic β cells secrete factors capable of inducing ribosomal proteins. Moreover, we found protein translation activity was increased in PC12 cells treated with Min6 cell culture supernatant. Based on these findings, we hypothesized the following possibilities: Under normal physiological conditions, pancreatic β cells may release humoral factors that help maintain the homeostasis of ribosomal components in neurons. However, in diabetic states, where pancreatic β cells fail to secrete sufficient humoral factors, decreasing ribosomal proteins may occur, decreasing protein synthesis, and ultimately leading to neuronal cell death. Further exploration of these hypotheses is warranted to elucidate their validity.

To further elucidate the proteins secreted by Min6 cells that attenuate Aβ-induced cell death, we conducted a comparative analysis of expressed genes between PC12 cells and Min6 cells using publicly available databases. KEGG pathway analysis about the secreted protein gene more expressed in Min6 than PC12 cells showed that genes of proteins that activate the PI3K–AKT signal pathway were significantly enriched. PI3K–AKT–mTOR signal pathway is well known to be involved in ribosome biogenesis (24). One of the characteristics of Min6 cell culture supernatant is that it can up-regulate of ribosomal protein genes in PC12 cells. We hypothesized possibility that one of the secreted factor, which can up-regulate of ribosomal protein genes, would be PI3K–AKT–mTOR activators.

Among the observed growth factor contained at Min6 cell culture supernatant, which can induce PI3K–AKT signaling, we focused on FGFs. There have been previous studies on the relationship between FGFs and AD (23), and several reports have shown the effectiveness of FGFs in treating AD. FGF1 promotes the growth of neurites damaged by Aβ (40). FGF18 improves streptozocin-induced cognitive impairment, reduces Aβ accumulation, and attenuates neuronal damage such as nuclear pyknosis and apoptosis in rat brain (41). FGF21 suppresses Aβ_1–42_-induced cell inflammation and apoptotic death in SH-SY5Y neuroblastoma cells (42). However, as far as we know, the relation between FGF23 and AD has not been reported.

FGF23 belongs to the endocrine FGF family (27). Previous studies have reported that FGF23 activates the PI3K–AKT signaling pathway and reduces the cell apoptosis rate of H_2_O_2_-stimulated neurons (43). Our findings show that FGF23 is expressed in Min6 cells, but not in PC12, SH-SY5Y, or U251 cells (Fig. 6). Expression of FGF receptor has been reported in PC12 cell line (44), and our findings show that recombinant FGF23 inhibits cell death induced by Aβ (Fig. 6).

In conclusion, our findings provide a novel insight by which secreted factors from pancreatic β cells induce ribosomal components in neuronal cells. Further analysis revealed that one of these factors, FGF23, is a novel neuroprotective factor capable of alleviating Aβ toxicity, highlighting one of the mechanisms of complex pathways underlying neuroprotection against AD.

## Materials and methods

### Materials

The human Aβ_1–42_ and Aβ_25–35_ peptides were obtained from the Peptide Institute (Osaka, Japan).

### Cell culture

*Mus musculus* pancreatic β cell line Min6 was cultured in Dulbecco's modified Eagle's medium (DMEM) containing 10% fetal calf serum, 25mM glucose, 50 μM β-mercaptoethanol, and 1% antibiotic–antimycotic mixed stock solution. The PC12 cell line was cultured in Roswell Park Memorial Institute medium containing 5% fetal calf serum (FCS), 10% horse serum, and 1% antibiotic–antimycotic mixed stock solution. U251 cell line was cultured in DMEM containing 10% FCS and 1% antibioticantimycotic mixed stock solution. The SH-SY5Y cell line was cultured in DMEM containing 10% FCS. All cell lines were incubated at 37 °C in 5% CO_2_.

### Cell treatment

Aβ_1–42_ (Peptide Institute) was dissolved in 0.02% NH_3_ at 200 μM. PC12 cells were seeded in a 24-well plate at a concentration of 16 × 10^4^ cells/mL in neurobasal medium containing a 2% B-27 supplement with antioxidants. Min6 cells were cultured in neurobasal medium containing a 2% B-27 supplement without antioxidants for 24 h at 37 °C. PC12 cells were treated with Min6 cell culture supernatant and 5 µM Aβ_1–42_ for 48 h at 37 °C.

For the glucose test, Min6 cells were cultured in Krebs-Ringer HEPES (KRH) buffer containing 5 or 25 mM glucose for 4 h at 37 °C. After the incubation, Min6 cell culture supernatant was centrifuged using a 3K Amicon ultra filter at 2,500 rpm for 1.5 h and dissolved in neurobasal medium containing a 2% B-27 supplement antioxidant-free.

For the thermal stability test, Min6 cell culture supernatant was collected in a 2-mL tube and boiled for 10 min. Then, a B-27 supplement without antioxidants was added to obtain a final concentration of 2%. Cell culture medium of PC12 cells was replaced with the Min6 cell culture supernatant, and then, 5 µM Aβ_1–42_ was treated (48 h at 37 °C).

For the RNA-seq analysis, Aβ_25–35_ (Peptide Institute) was dissolved in neurobasal medium at 10 mM and incubated for 7 days at 37 °C (45). Then, PC12 cells were treated with Min6 cell culture supernatant with or without Aβ_25–35_ (20 µM) for 48 h.

FGF23 (PeproTech, USA) was dissolved at 0.5 mg/ml. PC12 cells were seeded at 1 × 10^5^ cells/mL concentration in a neurobasal medium containing a 2% B-27 supplement with antioxidants. After 24-h incubation at 37 °C, for LDH assay, PC12 cell culture was changed to a neurobasal medium containing a 2% B-27 supplement without antioxidants and PC12 cells were treated with 0.1, 1, 10, 100, or 1,000 ng/ml FGF23 and 20 µM Aβ_25–35_ for 48 h at 37 °C. For western blotting, PC12 cell culture was changed to a neurobasal medium and PC12 cells were treated with 100 ng/ml FGF23 for 48 h at 37 °C.

### LDH release assay

The LDH level was determined using a cytotoxicity detection kit (Roche Molecular Biochemical, Basel, Switzerland) according to the manufacturer's instructions. Absorbance was measured at 492 nm, and the percentage of cell death was calculated by measuring the ratio of LDH activity in the culture medium to the total LDH activity (i.e. [extracellular LDH]/[extracellular LDH + cellular LDH]).

### ELISA

After Min6 cells were incubated in KRH buffer, 100 μL of the supernatant was collected. The insulin level of the supernatant was measured using a mouse insulin ELISA kit (Morinaga, Japan) according to the manufacturer's instructions.

Min6 cell culture supernatant was collected and concentrated 5-fold using Nanosep Omega 3K (OD003C34, Pall, Japan). The Fgf23 level in the supernatant was measured using a mouse FGF23 ELISA kit (R&D Systems, USA) according to the manufacturer's instructions.

### RNA-seq and DEG identification

PC12 cells were stimulated with 20 µM Aβ_25–35_ with or without Min6 culture supernatant (48 h) and designed into four groups (Control: nontreatment of Aβ nor Min6 cell culture supernatant; Aβ: treatment with Aβ; Sup_Control: treatment with Min6 culture supernatant; and Sup_Aβ: treatment with Aβ and Min6 cell culture supernatant). After the treatment, cellular RNA was extracted using an RNeasy Mini Kit according to the manufacturer's instructions. The preparation of the RNA library, transcriptome sequencing using DNBSEQ-T7 (MGI), and analysis were conducted by Novogene Co., Ltd. (Beijing, China).

DEG analysis was performed using the DESeq2 R package (1.20.0). Genes with an adjusted *P*-value < 0.05 found by DESeq2 were assigned as DEGs.

### GO and KEGG enrichment analysis of DEGs

GO enrichment analysis of DEGs was implemented by the clusterProfiler R package, in which the gene length bias was corrected. GO terms with corrected *P*-values < 0.05 were considered significantly enriched with DEGs. The statistical enrichment of DEGs was also tested in KEGG (http://www.genome.jp/kegg/) pathways. An adjusted *P*-value < 0.05 was considered significant for all the enrichment analyses.

### Gene set enrichment analysis

GSEA was conducted using GSEA software (version 4.3.2). Gene set permutations were conducted with 1,000 random combinations for every analysis. Nominal *P*-values < 0.05 were significantly enriched.

### Western blotting

Ribosome translation activity was measured by puromycin labeling (46). PC12 cells were treated with Min6 cell culture supernatant or FGF23 for 48 h, and before 15 min of harvesting, 10 μg/mL puromycin was diluted in the PC12 culture. Total protein was extracted from cells lysed with radioimmunoprecipitation assay (RIPA) buffer (Nacalai Tesque, Japan) containing a protease inhibitor cocktail (Nacalai Tesque). After centrifugation at 15,000 rpm for 20 min at 4 °C, the protein concentration in the supernatant was measured using the bicinchoninic acid (BCA) protein assay kit (Takara, Japan). An equivalent of 4–8 μg/lane cellular protein was separated by sodium dodecyl sulfate–polyacrylamide gel electrophoresis and electrotransferred onto a polyvinylidene fluoride membrane. After blocking with Blocking One (Nacalai Tesque) for 30 min, the membranes were probed with primary antibody at 4 °C overnight. Antibody against puromycin (MABE343, Sigma) was diluted 1:25,000 and β-actin (A5441, Sigma) was diluted 1:5,000 in Can Get Signal 1 (Toyobo, Japan), followed by incubation with the secondary antibody diluted 1:5,000 in Can Get Signal 2 (Toyobo) at room temperature for 1 h. The protein bands were visualized with ECL Prime Western Blotting System (Cytiva, USA), and images were captured with a Vilber Bio Imaging FUSION SOLO.7S.EDGE (M&S Instruments Inc., Japan). ImageJ software version 1.53 was used for quantitative analysis of the immunoreactive bands.

### Identification of genes using a database

The RNA-seq dataset of PC12 cells (GEO accession ID: GSE126087 (47)) and Min6 cells (GEO accession ID: GSE132072 (48)) was acquired from the GEO database (https://www.ncbi.nlm.nih.gov/geo/). The secreted proteins list was edited from The Human Protein Atlas. The “DEseq2” package in RStudio software (version 4.3.2) was used to select DEGs with |log_2_ fold change| > 1 and |adj. *P*-value | < 0.05. The statistical enrichment of DEGs was tested in KEGG (http://www.genome.jp/kegg/) pathways. An adjusted *P*-value < 0.05 was considered significant for all the enrichment analyses.

### Quantitative real-time PCR

After corresponding treatment PC12 cells, total RNA was extracted utilizing RNeasy mini kit (Qiagen, USA). Total RNA was reverse-transcribed into cDNA utilizing ReverTra Ace qPCR RT Master Mix (Toyobo) in accordance with the manufacturer's instructions. SYBR Green qPCR Master Mix (Thermo Scientific, USA) was used to produce quantitative real-time PCR (qRT-PCR), which was then run in triplicate and evaluated using a QuantStudio 1 qPCR system (Thermo Scientific). The *C*_t_ value was used to standardize the relative expression levels of target genes by using GAPDH as a reference gene. The following primers were used for qRT-PCR: mouse_FGF23 forward 5′-CACTGCTAGAGCCTATCC-3′ and reverse 5′-CACTGTAGATGGTCTGATGG-3′ (49); rat_FGF23 forward 5′-GCAACATTTTTGGATCGTATCA-3′ and reverse 5′-GATGCTTCGGTGACAGGTAGA-3′ (50); human_FGF23 forward 5′- TGCTGGCTTTGTGGTGATTA-3′ and reverse 5′-TTCTCCGGGTCGAAATAGTG-3′ (51); human_FGF1 forward 5′-TATACGGCTCACAGACACC-3′ and reverse 5′-TCTCTGCATGCTTCTTGGA-3′ (52); mouse_FGF1 forward 5′-GTTGTGATCTCCCCTTCAGC-3′ and reverse 5′-CGGACTTCATTCCCGTCTT-3′ (53); rat_FGF1 forward 5′-GCAAGGTTTTGGTGCTTACC-3′ and reverse 5′-TCGATGGTGCGTTCAAGAC-3′ (54); human_FGF18 forward 5′-TGCTTCCAGGTACAGGTGCT-3′ and reverse 5′-GCTGCTTACGGCTCACATCG-3′ (55); mouse_FGF18 forward 5′-AAGTCCGGATCAAGGGCAAG-3′ and reverse 5′-CATCAGGGCCGTGTAGTTGT-3′ (56); rat_FGF18 forward 5′-TGCGCTTGTACCAGCTCTAC-3′ and reverse 5′-CACTCCTTGCTAGTACCATC-3′ (57); mouse and rat_GAPDH forward 5′-AAACCCATCACCATCTTCCAG-3′ and reverse 5′-AGGGGCCATCCACAGTCTTCT-3′; human_GAPDH forward 5′-GAAGGTGAAGGTCGGAGTCA-3′ and reverse 5′-GAAGATGGTGATGGGATTTCC-3′.

### Statistical analysis

The results were expressed as mean ± SE. Statistical analyses were performed using the Student's t test, paired t test, Tukey–Kramer test, or Dunnett's test. We considered the data to be statistically significant when *P*-values were <0.05.

## Supplementary Material

pgae542_Supplementary_Data

## Data Availability

The data used in this study are available in the DNA Data Bank of Japan under the accession number PRJDB18107.
