# Supplementary material for: Pancreatic β cell-secreted factor FGF23 attenuates Alzheimer's disease-related amyloid β-induced neuronal death
Source: PNAS Nexus. 2025 Jan 28;4(1):pgae542. doi: 10.1093/pnasnexus/pgae542 (PMC11773612; doi:10.1093/pnasnexus/pgae542)
Supplement: pgae542_Supplementary_Data [file pgae542_supplementary_data.zip › PNASNEXUS-PNASNEXUS-2024-00780-TRR-s04.docx]

**Supplementary Figure 1. Pancreatic β cell culture supernatant upregulates ribosomal protein genes in PC12 neuronal cells.** (A) Volcano plot of differentially expressed genes (DEGs) in PC12 cells at Min6 cell culture supernatant-treated samples (Sup_Control) v.s. control samples (Control) (B) GO analysis of DEGs in PC12 at Sup_Control v.s. Control. (C) KEGG pathway analysis of DEGs in PC12 at Sup_Control v.s. Control**.**

**Supplementary Figure 2. Expression levels of FGFs.**

Expression levels of (A) *FGF1*, (B) *FGF18*, and (C) *FGF21* mRNA in Min6, PC12, SH-SY5Y, and U251 cells. Mean ± SE, n=3.

**Supplementary Figure 3. Translational activity level of PC12 cells.**

PC12 cells were treated with FGF23 (100 ng/ml). (A) Representative images of Western blots measuring translational activity. (B) Quantitative Western Blot Analysis of translational activity**.** Mean ± SE, n=4. **P <* 0.05. Paired *t*-test.
