# Supplementary figures and images for: Pancreatic β cell-secreted factor FGF23 attenuates Alzheimer's disease-related amyloid β-induced neuronal death
Source: PNAS Nexus. 2025 Jan 28;4(1):pgae542. doi: 10.1093/pnasnexus/pgae542 (PMC11773612; doi:10.1093/pnasnexus/pgae542)

Supplementary Figure. 1

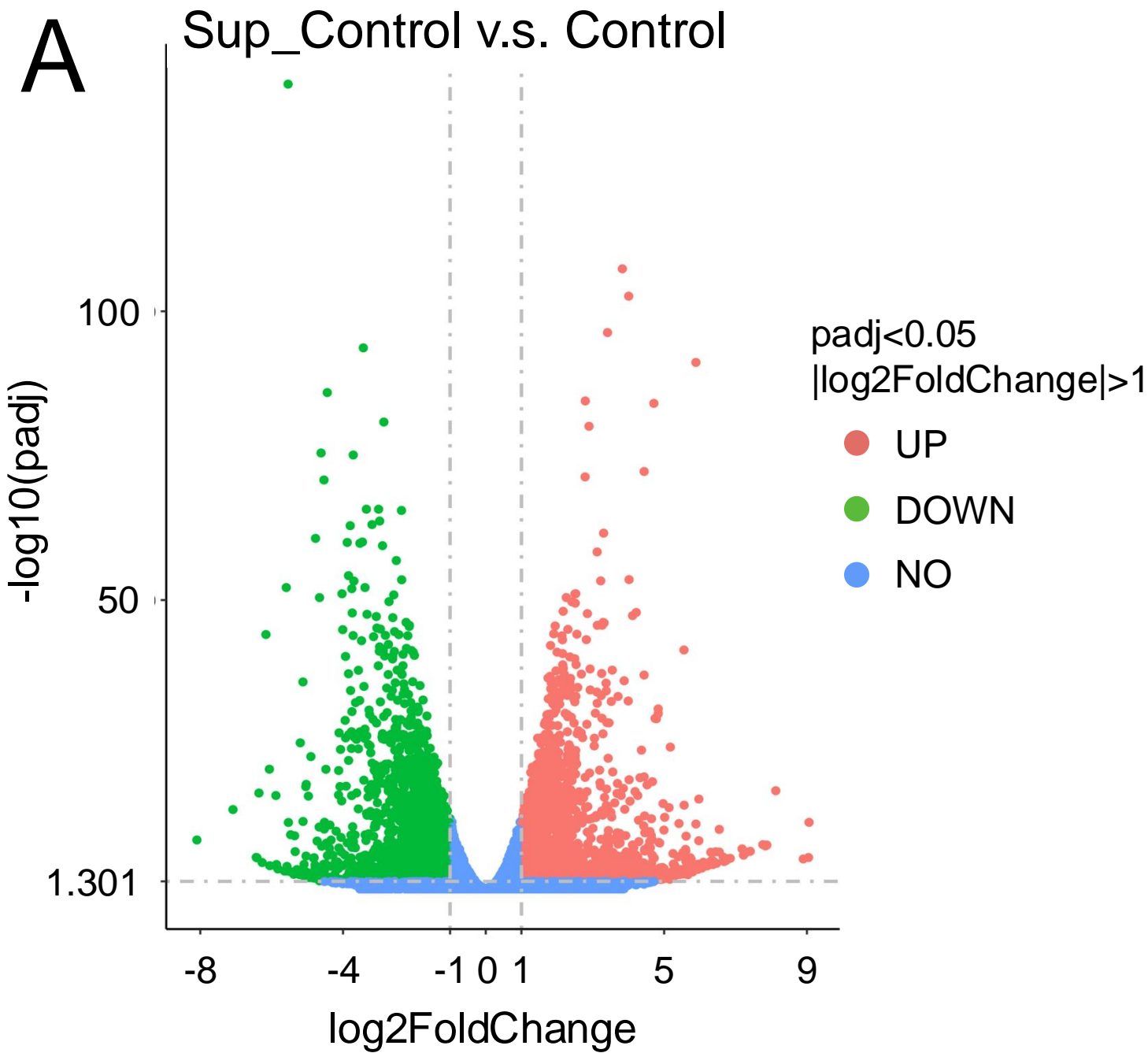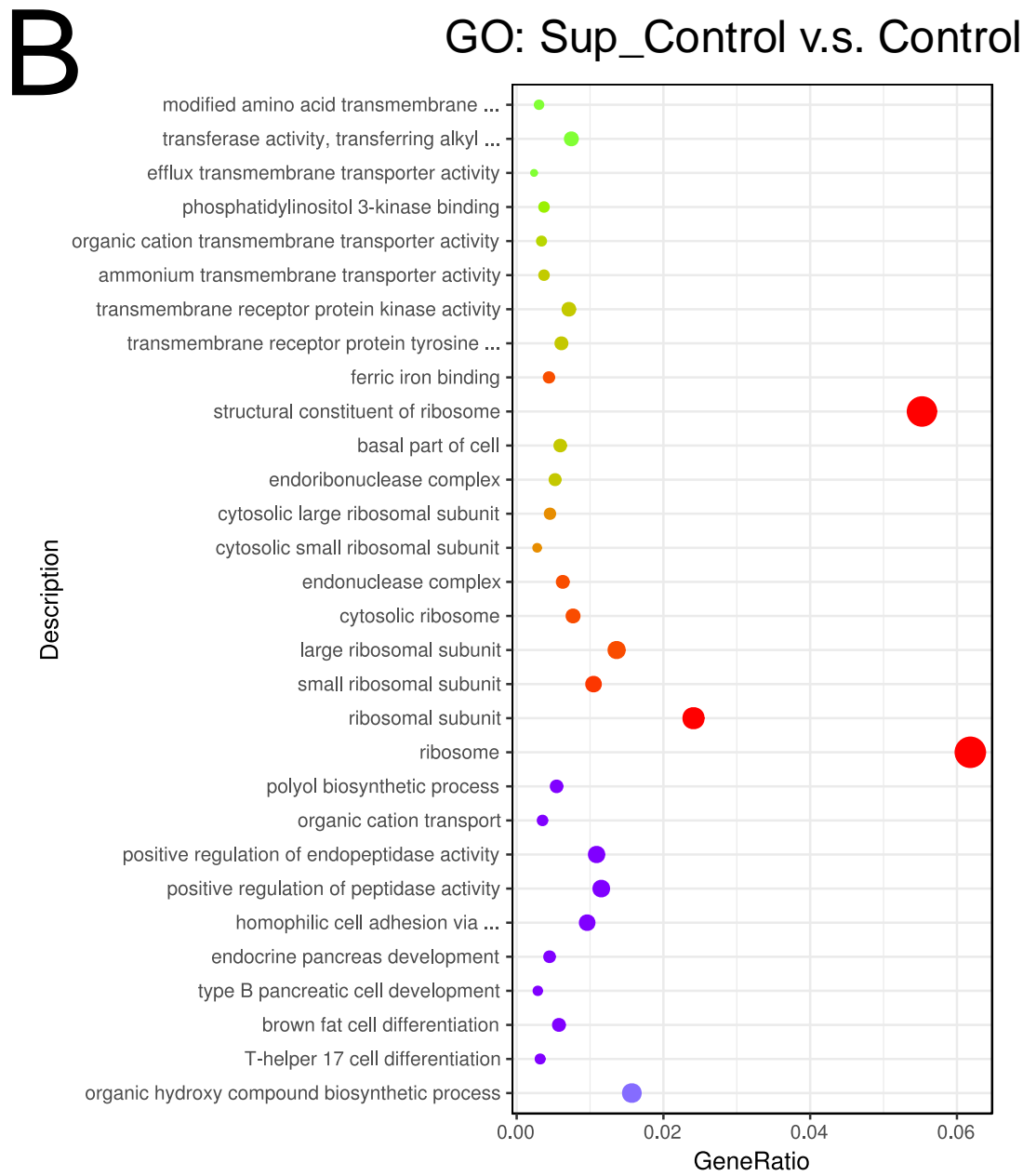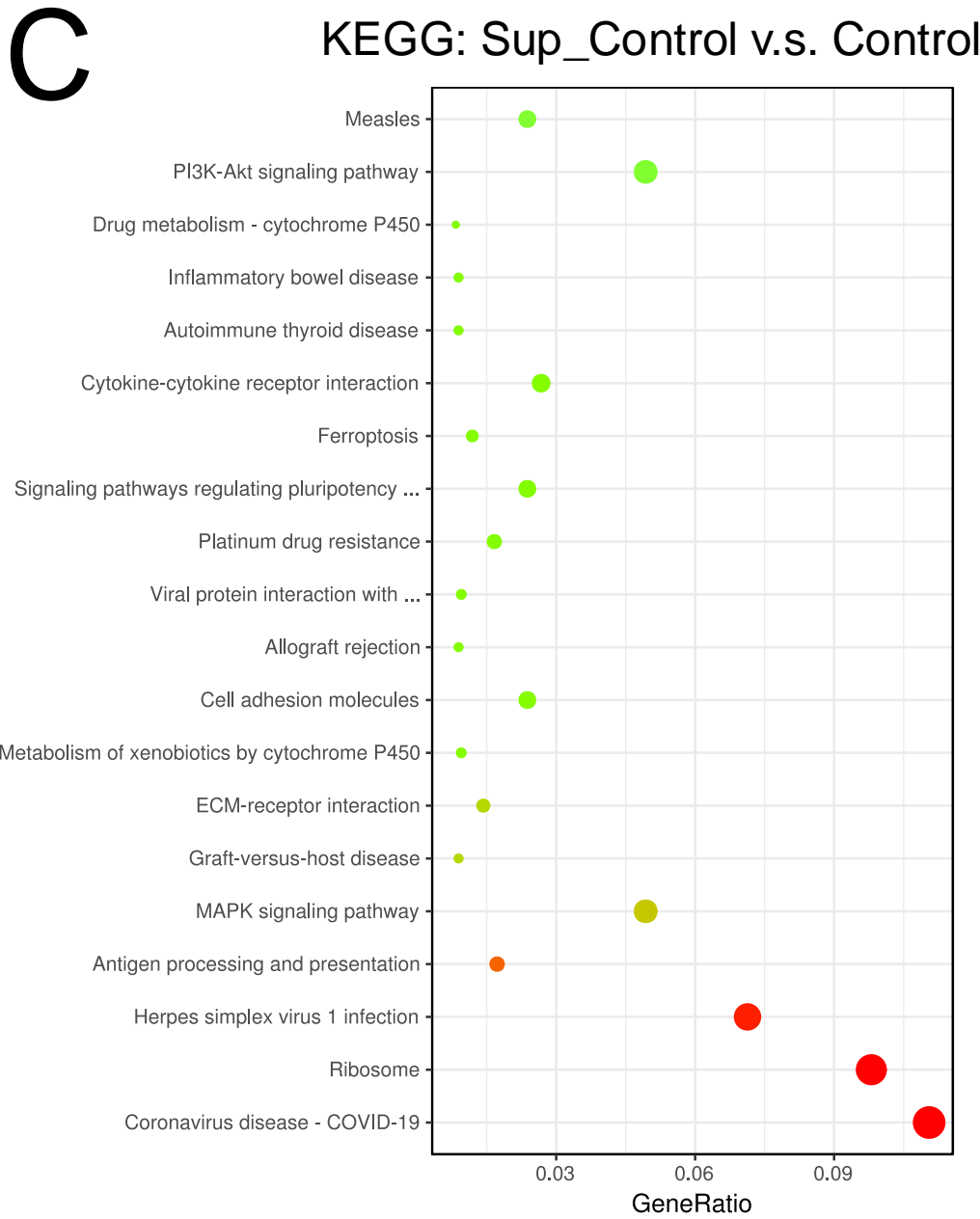

Supplement: pgae542_Supplementary_Data [file pgae542_supplementary_data.zip › PNASNEXUS-PNASNEXUS-2024-00780-TRR-s01.pdf]

Supplementary Figure. 2

A

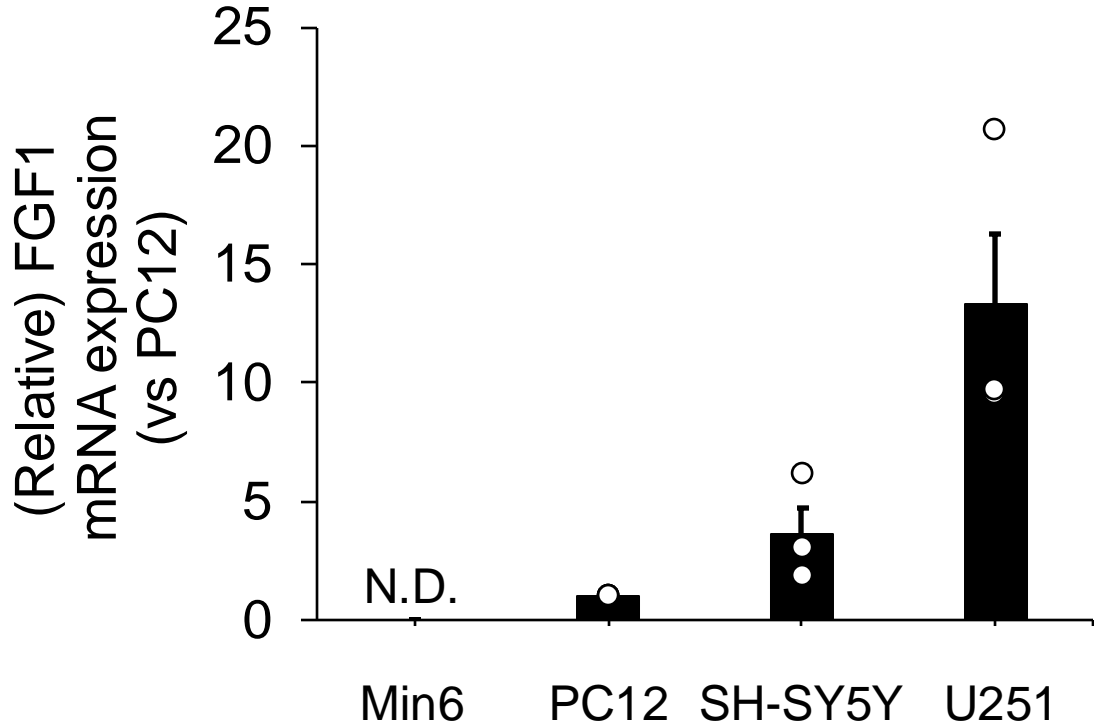

B

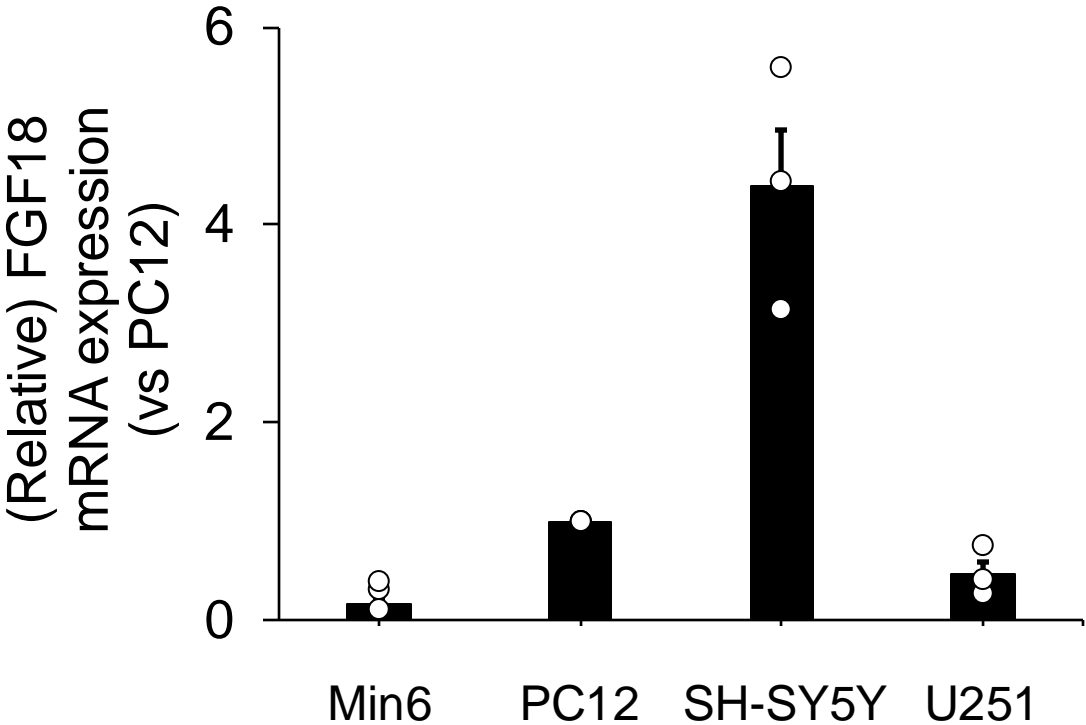

C

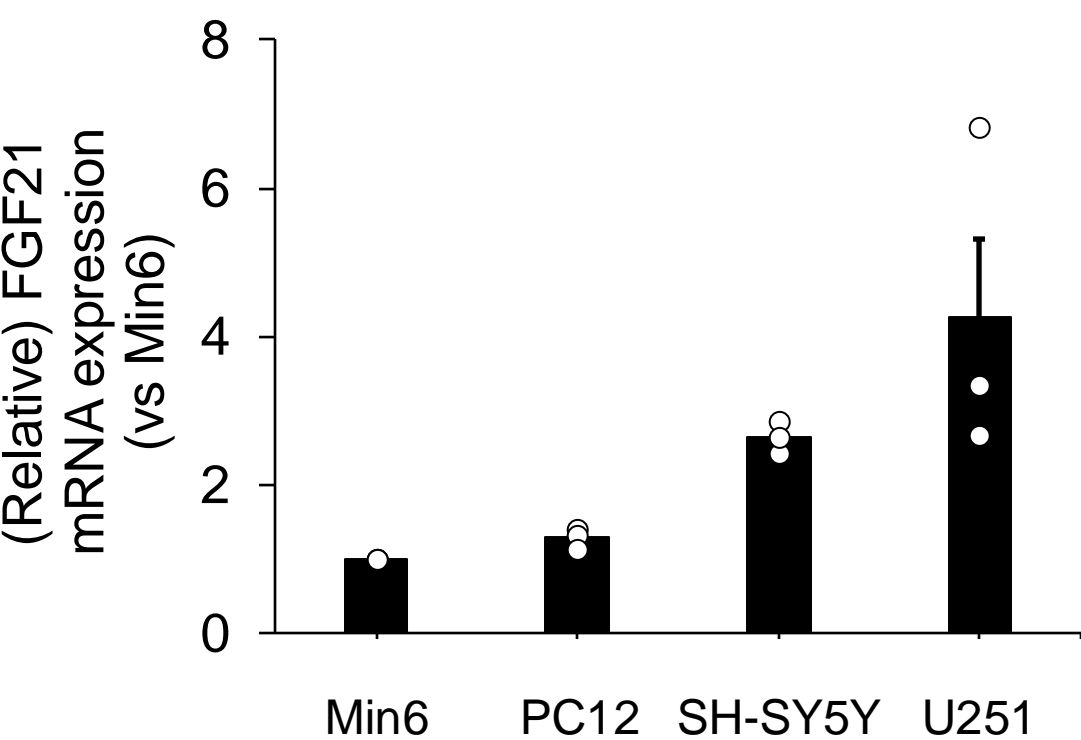

Supplement: pgae542_Supplementary_Data [file pgae542_supplementary_data.zip › PNASNEXUS-PNASNEXUS-2024-00780-TRR-s02.pdf]

# Supplementary Figure. 3

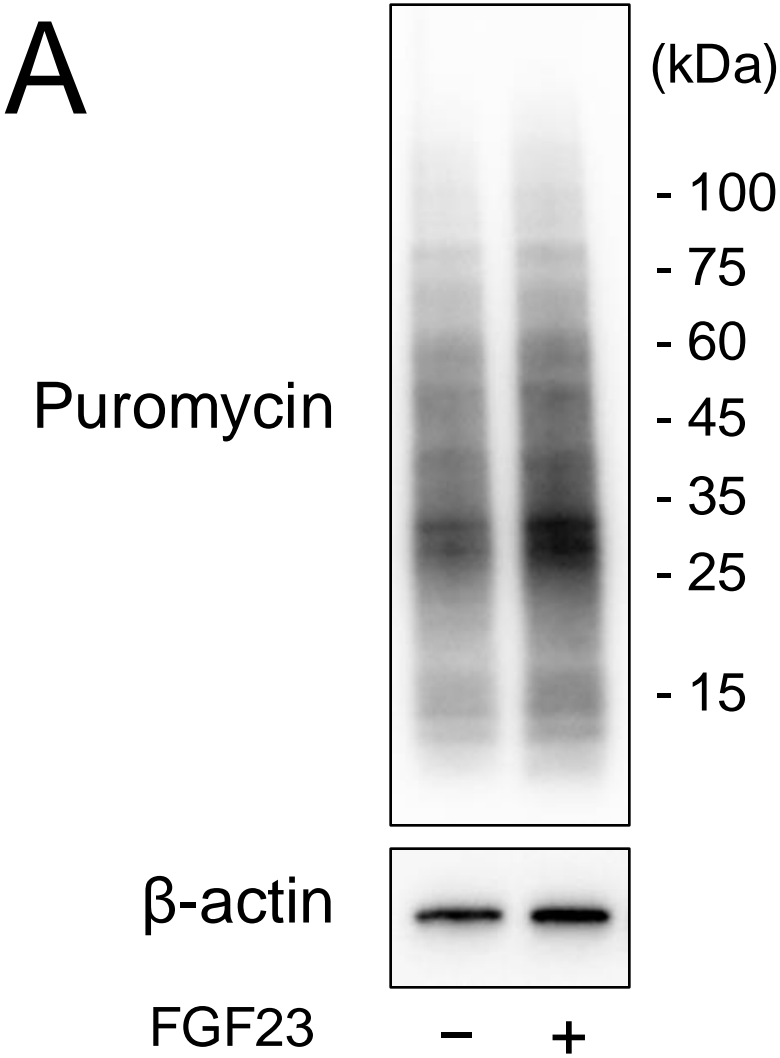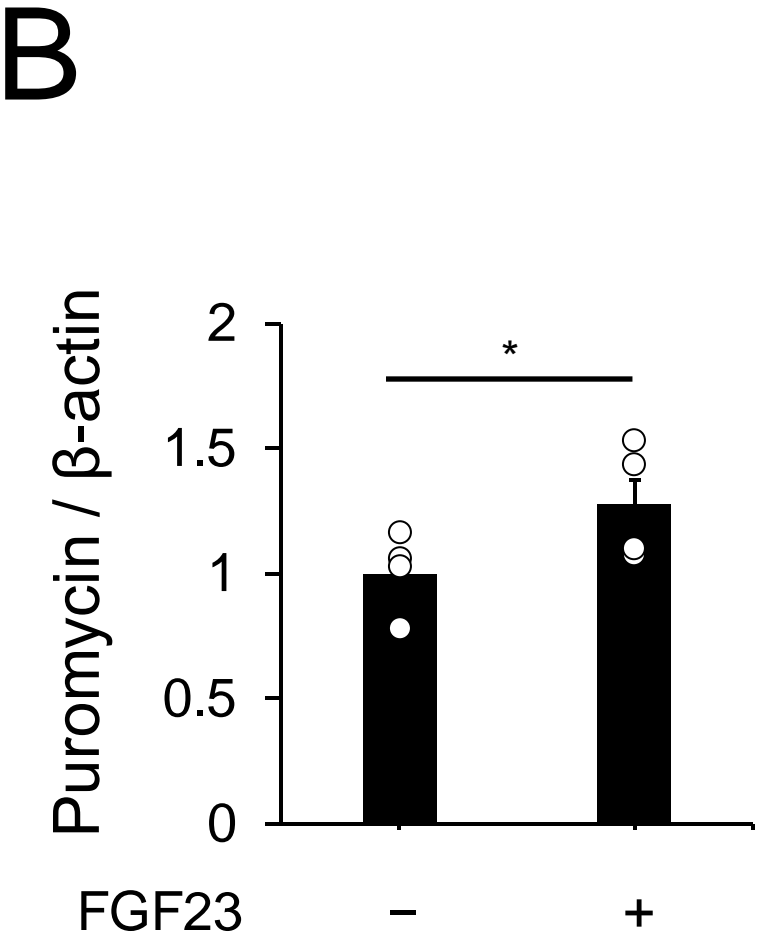

Supplement: pgae542_Supplementary_Data [file pgae542_supplementary_data.zip › PNASNEXUS-PNASNEXUS-2024-00780-TRR-s03.pdf]
